# Pro-Inflammatory Implications of 2-Hydroxypropyl-β-cyclodextrin Treatment

**DOI:** 10.3389/fimmu.2021.716357

**Published:** 2021-08-20

**Authors:** Tom Houben, Tulasi Yadati, Robbin de Kruijf, Marion J. J. Gijbels, Joost J. F. P. Luiken, Marc van Zandvoort, Dimitris Kapsokalyvas, Dieter Lütjohann, Marit Westerterp, Jogchum Plat, David Leake, Ronit Shiri-Sverdlov

**Affiliations:** ^1^Departments of Genetics and Cell Biology, School of Nutrition and Translational Research in Metabolism (NUTRIM), University of Maastricht, Maastricht, Netherlands; ^2^School for Oncology and Developmental Biology GROW, School of Nutrition and Translational Research in Metabolism (NUTRIM) and School for Cardiovascular Diseases CARIM Maastricht University, Maastricht, Netherlands; ^3^Institute for Molecular Cardiovascular Research IMCAR, Rheinisch-Westfälische Technische Hogeschool (RWTH) Aachen University, Aachen, Germany; ^4^Institute of Clinical Chemistry and Clinical Pharmacology, University Hospital Bonn, Bonn, Germany; ^5^Department of Pediatrics, University of Groningen, University Medical Center Groningen, Groningen, Netherlands; ^6^Department of Nutrition and Movement Sciences, School for Nutrition and Translational Research in Metabolism (NUTRIM), Maastricht University, Maastricht, Netherlands; ^7^School of Biological Sciences, University of Reading, Health and Life Sciences Building, Whiteknights, Reading, United Kingdom

**Keywords:** 2-hydroxypropyl-β-cyclodextrin, metabolic inflammation, cholesterol, hepatic inflammation, macrophage

## Abstract

Lifestyle- and genetically induced disorders related to disturbances in cholesterol metabolism have shown the detrimental impact of excessive cholesterol levels on a plethora of pathological processes such as inflammation. In this context, two-hydroxypropyl-β-cyclodextrin (CD) is increasingly considered as a novel pharmacological compound to decrease cellular cholesterol levels due to its ability to increase cholesterol solubility. However, recent findings have reported contra-indicating events after the use of CD questioning the clinical applicability of this compound. Given its potential as a therapeutic compound in metabolic inflammatory diseases, in this study, we evaluated the inflammatory effects of CD administration in the context of cholesterol-induced metabolic inflammation *in vivo* and *in vitro*. The inflammatory and cholesterol-depleting effects of CD were first investigated in low-density lipoprotein receptor knockout (*Ldlr^-/^*) mice that were transplanted with *Npc1^nih^* or *Npc1^wt^* bone marrow and were fed either regular chow or a high-fat, high-cholesterol (HFC) diet for 12 weeks, thereby creating an extreme model of lysosomal cholesterol-induced metabolic inflammation. In the final three weeks, these mice received daily injections of either control (saline) or CD subcutaneously. Subsequently, the inflammatory properties of CD were investigated *in vitro* in two macrophage cell lines and in murine bone marrow-derived macrophages (BMDMs). While CD administration improved cholesterol mobilization outside lysosomes in BMDMs, an overall pro-inflammatory profile was observed after CD treatment, evidenced by increased hepatic inflammation *in vivo* and a strong increase in cytokine release and inflammatory gene expression *in vitro* in murine BMDMs and macrophages cell lines. Nevertheless, this CD-induced pro-inflammatory profile was time-dependent, as short term exposure to CD did not result in a pro-inflammatory response in BMDM. While CD exerts desired cholesterol-depleting effects, its inflammatory effect is dependent on the exposure time. As such, using CD in the clinic, especially in a metabolic inflammatory context, should be closely monitored as it may lead to undesired, pro-inflammatory side effects.

## Introduction

As one of the most important steroid alcohols of the human body, cholesterol carries a number of essential functions ranging from acting as a key structural component of cell membranes (1) to serving as precursor for bile acids, steroid hormones and vitamin D metabolites (2). Due to this key cellular function of cholesterol, a highly coordinated extra- and intracellular transport system maintains cholesterol homeostasis at a whole body as well as at a cellular level (3, 4). Evidently, deficiencies in this transport system leading to a disproportionate cellular supply of cholesterol result in severe pathogenic profiles. At one hand, these deficiencies can be induced *via* mutations in genes encoding for receptors and/or enzymes involved with cholesterol homeostasis. Examples of such disorders include Wolman disease (5) and the Niemann-Pick diseases (6) (both lysosomal storage disorders) as well as familial hypercholesterolemia (7). Though these diseases are rather rare, their pathogenesis is often severe and sometimes results in premature death. Besides genetic predisposition, another and more prevalent manner that influences cholesterol homeostasis is *via* excessive overnutrition, leading to metabolic syndrome and its associated diseases (8). In this context, excessive cholesterol accumulates at locations such as the vessel wall (atherosclerosis) and the liver (non-alcoholic fatty liver disease (NAFLD)) subsequently triggering resident macrophages to evolve into foam cells characterized by lysosomal cholesterol accumulation that induces an undesired inflammatory response, also referred to as metabolic inflammation (4, 9). This inflammatory response is an antecedent for more severe consequences both in the context of atherosclerosis (heart attack, stroke, peripheral vascular disease (10)) as well as NAFLD (advanced liver diseases such as cirrhosis and hepatocellular carcinoma (9)). Taken the prevalence and the severity of these disorders into consideration, efficient therapeutic options aimed at reducing cellular cholesterol levels are needed.

A straightforward and successfully applied approach to reduce cholesterol levels is *via* pharmacological approaches, which include the use of statins (11), fibrates (12) and more recently antibodies targeting proprotein convertase subtilisin-kexin type 9 (PCSK9) (13). However, substantial reduction of plasma cholesterol levels cannot be guaranteed for all patients (11), requiring alternative ways to improve cholesterol homeostasis. Relevantly, while the aforementioned interventions focus on reducing plasma cholesterol levels, a relative new approach aims at mobilizing cholesterol at the site of accumulation. This has led to the proposition to use 2-hydroxypropyl-β-cyclodextrin (CD), a substance initially used to solubilize lipophilic pharmaceutical agents, as a compound to treat cholesterol accumulation. Indeed, CD mobilized cholesterol from foam cells (14), promotes atherosclerosis regression (15), improved hepatic cholesterol metabolism (16), reduced lysosomal size of neural stem cells derived from Wolman disease patients (17) and is under evaluation in advanced human clinical trials for Niemann-Pick disease, type C1 (NPC1) (18). In contrast to these beneficial effects, CD causes massive damage to all cells of the developing (19) and some cells of the adult auditory system (20). Moreover, while dietary supplementation of β-cyclodextrin in hypercholesterolemic rats improved lipid metabolism, it concomitantly produced hepatotoxic effects characterized by increased plasma aminotransferase levels (21). Together, though the use of CD has clearly shown its benefits, the previous observations question whether this compound can be directly used in the clinic. Given its potential as therapeutic compound in metabolic inflammatory diseases, in this study, we therefore investigated the inflammatory aspects of CD administration in the context of cholesterol-induced metabolic inflammation *in vivo* and *in vitro*.

For this purpose, we investigated the inflammatory and cholesterol-depleting properties of CD *in vivo* and in a series of *in vitro* experiments for metabolic inflammation. First, we analyzed the effect of CD injection in low-density lipoprotein receptor knockout (*Ldlr^-/-^*) mice transplanted with *Npc1^nih^* or *Npc1^wt^* bone marrow on a high-fat, high-cholesterol (HFC) diet. Due to the absence of LDL receptors, and following an HFC diet, *Ldlr^-/-^* mice are characterized by high plasma LDL levels, mimicking diet-induced dyslipidemia and serving as an excellent animal model for metabolic diseases. Furthermore, *Npc1^nih^* bone marrow-transplanted mice feature a dysfunctional NPC1 protein in their immune cells, due to a deleterious frameshift mutation in the *Npc1* allele, leading to lysosomal cholesterol buildup and a pro-inflammatory state. As such, chimeric *Npc1^nih^* transplanted (*Npc1^nih^*-tp) *Ldlr^-/-^* mice given an HFC diet constitute an extreme state of lysosomal cholesterol-induced metabolic inflammation (22). Next, as CD is able to mobilize lysosomal cholesterol, we assessed the ability of CD to influence lysosomal size of metabolically challenged bone marrow-derived macrophages (BMDM) by employing confocal microscopy. Finally, in a series of *in vitro* experiments we investigated the inflammatory properties of CD in more detail.

## Materials and Methods

### Mice, Bone Marrow Transplant, Diet and Injections

Throughout the study, mice were housed under standard conditions and had unlimited access to food and water, unless explicitly mentioned otherwise. For one week prior to and up to four weeks after bone marrow transplantation, *Ldlr^-/-^* mice were housed in filter-top cages and received antibiotics diluted in drinking water to prevent infections following immunosuppression (Neomycin, 100 mg/l, Gibco, Breda, the Netherlands; 6*10^4^ U/L polymycin B sulfate). Six weeks old bone marrow donors *Npc1^nih^* and *Npc1^wt^* mice were derived from heterozygous founders of a C57BL/6 genetic background. Genotype of *Npc1^nih^* and *Npc1^wt^* mice was determined as previously described (23). On the day of the bone marrow transplant, *Npc1^nih^* and *Npc1^wt^* littermates were sacrificed *via* CO_2_ inhalation and their bone marrows were isolated. One day before and on the day of the bone marrow transplant, *Ldlr^-/-^* mice were subjected to six Gray of γ-radiation, thus having received 12 Gray of γ-radiation before receiving 1*10^7^ bone marrow cells collected from *Npc1^wt^* or *Npc1^nih^* mice *via* intravenous injection. After ten weeks of recovery, transplanted mice were placed on a high-fat, high-cholesterol (HFC) diet (24) for twelve weeks. In the final three weeks (based on (16, 25)), mice received daily, subcutaneous injections of control (saline) or 2-hydroxypropyl-β-cyclodextrin (1800 mg/kg body weight; CD, Sigma-Aldrich), creating four experimental groups: (1) *Npc1^wt^*-tp NaCl (n = 6), (2) *Npc1^wt^*-tp CD (n = 9), (3) *Npc1^nih^*-tp NaCl (n = 6) and (4) *Npc1^nih^*-tp CD (n = 9). A schematic overview of the experimental set-up is provided in **Supplementary Figure S1**. All experiments were performed according to Dutch laws and approved by the Animal Experiment Committee of Maastricht University.

Upon sacrifice, all tissues were isolated and snap-frozen in liquid nitrogen and stored at -80°C or fixed in 4% formaldehyde/PBS. The collection of blood and tissue specimens, biochemical determination of lipids in plasma, RNA isolation, cDNA synthesis and qPCR were determined as described previously (26, 27). Primer sequences for genes are listed in **Supplementary Table 1**. Hepatic sterol content was determined by gas-liquid chromatography-mass spectroscopy, as described elsewhere (28).

### Immunohistochemistry

Frozen liver sections (7 µm) were fixed in acetone and blocked for endogenous peroxidase by incubation with 0.25% of 0.03% H_2_O_2_ for 5 minutes. Primary antibodies used were against hepatic macrophages (1:100 rat anti-mouse CD68, clone FA11), infiltrated macrophages and neutrophils (1:500 rat anti-mouse Mac-1 (M1/70)) and neutrophils (1:100 rat anti-mouse NIMP (Ly6G)). 3-Amino-9-ethylcarbazole (Vector laboratories, CA, USA) was applied as color substrate and hematoxylin for nuclear counterstain. Sections were enclosed with Faramount aqueous mounting medium. Additional information concerning the immunostainings is also described elsewhere (29).

Pictures were taken with a Nikon digital camera DMX1200 and ACT-1 v2.63 software (Nikon Instruments Europe, Amstelveen, The Netherlands). Infiltrated macrophages and neutrophil cells (Mac-1^+^) and neutrophils (NIMP) were counted by two blinded researchers in six microscopical views (original magnification, 200x) and were indicated as number of cells per square millimeter (cells/mm^2^). Hepatic macrophages (CD68) were counted in six microscopical views (original magnification, 200x) and indicated as the percentage of CD68 positive area (Adobe Photoshop CS2 v.9.0.).

### Fluorescence Confocal Microscopy and Quantification of Lysosomes Categorized by Size

#### LAMP1 Staining

For assessing lysosomal size, we performed fluorescence confocal microscopy by staining for the lysosomal membrane marker lysosomal-associated membrane protein 1 (LAMP-1) followed by quantification of the number of lysosomes based on size. For this staining, fresh BMDM were fixed in paraformaldehyde (4%) and permeabilized in Triton-X (0.1%)/BSA (0.2%) solution. BMDM were incubated with the primary (1:100, rabbit polyclonal Lamp1, ab24170, Abcam, Cambridge, United Kingdom) and secondary (1:200, Alexa fluor 488 goat anti-rabbit IgG, A11008, Thermo Fisher Scientific, Waltham, Massachusetts, USA) antibodies and finally, sections were enclosed with glycerol mounting medium (DABCO-DAPI). Confocal pictures were taken with a LEICA DMI 4000 microscope (Leica microsystems, Wetzlar, Germany), providing 3D images of BMDM and their lysosomes.

#### Lysosomal Quantification

##### Step 1: Configuring and adapting the lysosomal pictures

For optimal quantification, one cell remained visible per purview. For this purpose, the original photo was cropped, and then configured into a TIFF file for further processing.

##### Step 2: Splitting the channels for nucleus and lysosomal quantification

In this study we made use of two fluorescent stainings either for the nucleus (DAPI) or lysosome (LAMP-1). To determine the nucleus to lysosomal distance, the color channels were bifurcated for better differentiation, creating a nucleus channel and lysosomal channel.

##### Step 3: Coordinates of the nucleus.

To determine the lysosome to nucleus distance we first determined the location of the nucleus in the 3D picture. Therefore, we analyzed the nucleus channel with the 3D ObjectCounter.

##### Step 4: Creating a mask of lysosome channel

To obtain the coordinates of the lysosomes, a mask was created by using the ObjectCounter. The mask was created by calculating the geometrical centers of the lysosomes (30). Threshold was determined before the picture was analyzed.

##### Step 5: Watershed

To discriminate between separated or combined lysosomes, the watershed method was used. The watershed method uses the mask to determine the centers of the masses (Parra et al.).

##### Step 6: Coordinates of the lysosomes

After the watershed, the Regions of interest (ROIs) were added to the 3D ROI Manager, where the volume and coordinates were calculated. To quantify the pictures in an efficient manner, a macro was created (**Supplementary Methods**).

##### Step 7: Calculating the distance

After the coordinates and volumes were obtained, the distance for each lysosome to the nucleus was calculated. For this we used the Pythagoras equation.

$$\mathrm{Distance}=\surd ({\left({\text{X}}_{\text{nucleus}}-\text{ X}{\text{l}}_{\text{ysosome}}\right)}^{2}+{\left({\text{Y}}_{\text{nucleus}}-\;{\text{Y}}_{\text{lysosome}}\right)}^{2}+{\left({\text{Z}}_{\text{nucleus}}-\;{\text{Z}}_{\text{lysosome}}\right)}^{2})$$

Next, we created a plugin-Macro for ImageJ to identify the amount and volume of lysosomes. Finally, the number of lysosomes was quantified and categorized them by size (< 0.1 µm^3^; 0.1-1 µm^3^; > 1 µm^3^) and expressed the number of lysosomes per size relative to the total amount of lysosomes present inside the BMDM. Representative videos were created using Z-stack images played in series and recorded as.AVI files which were trimmed using quick time movie player. DAPI (blue) represents nuclei and LAMP-1 (green) represents lysosomes.

### Statistical Analysis

Data were statistically analyzed by performing the unpaired t-test or the two-way ANOVA and Tukey’s *post hoc* test using GraphPad Prism software (version 6 for Windows, GraphPad Software Inc, San Diego, CA, U.S; www.graphpad.com). Data were expressed as the group mean and standard error of the mean.

## Results

### Administration of 2-Hydroxypropyl-β-cyclodextrin Mobilizes Hepatic Cholesterol in *Npc1^nih^*-tp *Ldlr^-/-^* Mice

*Npc1^nih^* bone marrow-transplanted mice feature a dysfunctional NPC1 protein in their immune cells, due to a deleterious frameshift mutation in the *Npc1* allele, leading to lysosomal cholesterol buildup and a pro-inflammatory state. As such, chimeric *Npc1^nih^* transplanted (*Npc1^nih^*-tp) *Ldlr^-/-^* mice given an HFC diet constitute an extreme state of lysosomal cholesterol-induced metabolic inflammation (22).

**Figure 1:**
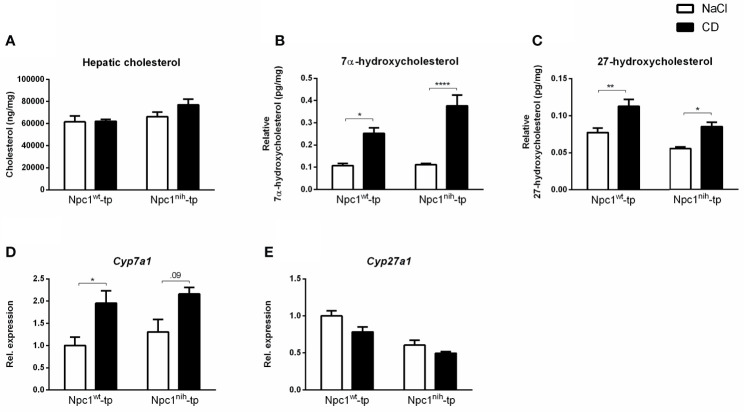
Hepatic lipid parameters. **(A)** Hepatic cholesterol levels of *Npc1^wt^*-tp and *Npc1^nih^*-tp mice on a HFC diet that received CD or saline treatment. **(B, C)** Hepatic levels of the cholesterol degradation products 7α-hydroxycholesterol and 27-hydroxycholesterol. **(D, E)** Hepatic gene expression analysis of *Cyp7a1* **(D)** and *Cyp27a1* **(E)**. n = 6-9 mice/group. Gene expression data are set relative to *Npc1^w^* ^t^-tp mice treated with saline. ^*^, ^**^ and ^****^ indicate *p* ≤ 0.05, 0.01 and 0.0001 resp.by means of two-way ANOVA followed by Tukey post-hoc analysis.

To confirm successful injection of 2-hydroxypropyl-β-cyclodextrin (CD) into the mice in the final three weeks of the experiments, plasma and hepatic lipid metabolism were profiled. CD-treated *Npc1^wt^*-tp *Ldlr^-/-^* mice showed reduced plasma triglycerides (**Table 1**), though this reduction was already apparent before the start of CD treatment (**Table 1**). While no effects were observed on plasma (**Table 1**) and hepatic (**Figure 1**) cholesterol levels, the ratios of the cholesterol oxidation products 7α-hydroxycholesterol to cholesterol and 27-hydroxycholesterol to cholesterol were both significantly increased in the livers of CD-treated *Npc1^nih^*- and *Npc1^wt^*-tp *Ldlr^-/-^* mice, suggesting increased mobilization of cholesterol (**Figures 1B, C**). Hepatic gene expression analysis of cytochrome P450 7A1 (*Cyp7a1)*, the enzyme responsible for the conversion of cholesterol into 7α-hydroxycholesterol, was concomitantly increased (**Figure 1D**), while sterol 27-hydroxylase (*Cyp27a1)* hepatic gene expression levels remained unaffected upon CD-treatment (**Figure 1E**). Concerning other non-cholesterol sterols, CD-treatment reduced hepatic cholestanol and desmosterol levels in CD-treated *Npc1^nih^*-tp *Ldlr^-/-^* mice (**Table 2**). No effects were observed on liver and spleen weight (**Supplementary Figures 2A, B**). Body weight over time seemed to be lower in *Npc1^nih^*-tp *Ldlr^-/-^* mice compared to *Npc1^wt^*-tp *Ldlr^-/-^* mice, but this difference was not significant (**Supplementary Figure 2C**). Overall, these findings indicate that CD-treatment mobilizes hepatic cholesterol, confirming successful administration of CD.

**Table 1:** Plasma total cholesterol and triglyceride levels.

	*Npc1^wt^*-tp		*Npc1^nih^*-tp	
	NaCl	Cyclo	NaCl	Cyclo
**Plasma cholesterol (mM)**				
T0	9.48 (± 0.86)	7.87 (± 0.73)	6.372 (± 0.30)	5.81 (± 0.69)
T9	148.15 (± 5.33)	118.3 (± 11.77)	66.68 (± 8.60)	80.72 (± 4.13)
T12	137.15 (± 4.73)	120.62 (± 9.68)	97.97 (± 6.61)	112.76 (± 8.48)
**Plasma triglycerides (mM)**				
T0	1.30 (± 0.11)	1.47 (± 0.17)	0.96 (± 0.11)	0.93 (± 0.11)
T9	5.57 (± 0.82)	2.99 (± 0.44)^**^	1.17 (± 0.25)	1.10 (± 0.19)
T12	3.18 (± 0.47)	1.88 (± 0.46)^***^	0.58 (± 0.13)	0.64 (± 0.11)

** and *** indicates p < 0.01 and 0.001 by means of two-way ANOVA followed by Tukey post-hoc analysis.

**Table 2:** Hepatic ratios of non-cholesterol sterols to cholesterol.

	*Npc1^wt^*-tp		*Npc1^nih^*-tp	
	NaCl	Cyclo	NaCl	Cyclo
**Absorption markers**				
*R_Sitosterol (pg/mg)*	0.05 (± 0.01)	0.05 (± 0.01)	0.13 (± 0.01)	0.12 (± 0.01)
*R_Campesterol (pg/mg)*	0.12 (± 0.01)	0.12 (± 0.01)	0.14 (± 0.03)	0.12 (± 0.02)
*R_Cholestanol (pg/mg)*	4.73 (± 0.25)	5.00 (± 0.16)	3.91 (± 0.16)	3.06 (± 0.11)*^a^*
**Cholesterol synthesis markers**				
*R_Lathosterol (ng/mg)*	0.49 (± 0.05)	0.56 (± 0.05)	0.33 (± 0.03)	0.28 (± 0.01)
*R_Desmosterol (ng/mg)*	0.29 (± 0.02)	0.30 (± 0.01)	1.17 (± 0.10)	0.84 (± 0.09)*^b^*
*R_Lanosterol (pg/mg)*	28.6 (± 2.2)	30.2 (± 2.0)	41.2 (± 1.1)	34.7 (± 1.9)

Data is given as mean ± standard error of the mean (S.E.M.).

^a,b^Indicates p < 0.05 and p < 0.01 respectively compared to NaCl-injected Npc1^nih^-tp mice.

All values are given as the ratio sterol to cholesterol.

### Administration of 2-Hydroxypropyl-β-cyclodextrin Increases Hepatic Inflammation and Fibrotic Markers in *Npc1^nih^*-tp *Ldlr^-/-^* Mice

**Figure 2:**
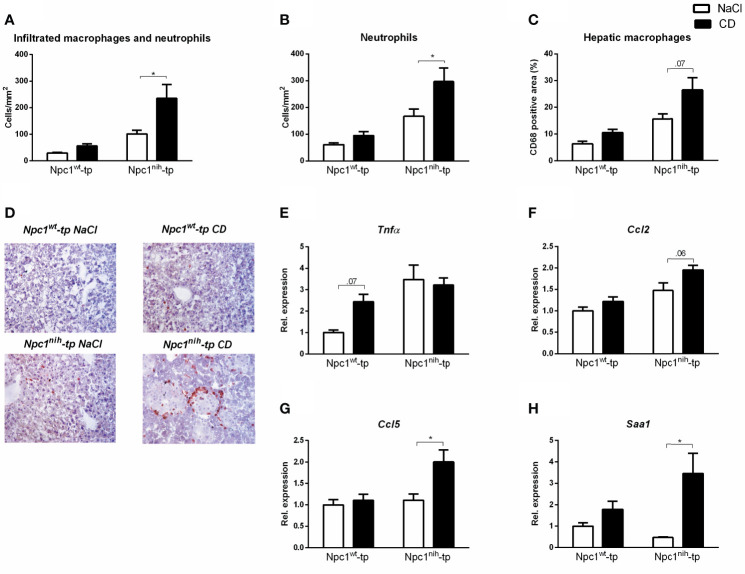
Parameters of hepatic inflammation. **(A–C)** Liver sections were stained for infiltrating macrophages and neutrophils (Mac-1), neutrophils (NIMP) and hepatic macrophages (CD68) and subsequently quantified by counting (Mac-1 and NIMP) or by assessing the positive area of the staining (CD68). **(D)** Representative images of liver sections stained for infiltrated macrophages and neutrophils (Mac-1). **(E–H)** Hepatic gene expression analysis of *Tnfα*, *Ccl2, Ccl5* and *Saa1*. Gene expression data were set relative to control-treated *Npc1^wt^*-tp mice. ^*^ indicates *p* ≤ 0.05 by means of two-way ANOVA followed by Tukey post-hoc analysis.

After we confirmed successful administration of CD, we further evaluated the impact of three-week CD treatment on hepatic inflammation by staining hepatic cryosections for the inflammatory markers Mac-1 (infiltrated macrophages and neutrophils; directed against Cd11b), NIMP (neutrophils) and CD68 (macrophages) (**Figures 2A–C**). While CD treatment only induced a minor non-significant increase for these inflammatory markers in *Npc1^wt^*-tp *Ldlr^-/-^* mice, this increase was more pronounced in *Npc1^nih^*-tp *Ldlr^-/-^* mice (**Figures 2A, D**). This pro-inflammatory effect of CD was confirmed by histological scoring of HE-staining of livers of the *Npc1^nih^*-tp *Ldlr^-/-^* mice (**Supplementary Figure S3**). To confirm these histological data, hepatic gene expression for the inflammatory markers tumor necrosis factor alpha (*Tnfα*), chemokine (C-C motif) ligand 2 (*Ccl2*), chemokine (C-C motif) ligand 5 (*Ccl5*) and serum amyloid A1 (*Saa1*) was analyzed. In line with the histological data, CD treatment increased the expression of these hepatic inflammatory markers in the *Npc1^wt^*-tp or in the *Npc1^nih^*-tp group (**Figures 2E–H**). Moreover, the hepatic fibrotic markers matrix metallopeptidase 9 (*Mmp9*) and plasminogen activator inhibitor-1 (*Pai-1*) were concomitantly increased in CD-treated *Npc1^nih^*-tp *Ldlr^-/-^* mice, while no significant changes were observed in transforming growth factor beta (*Tgfβ*) and tissue inhibitor of metalloproteinase (*Timp1*) (**Supplementary Figure S4**). Together, these findings indicate that three-week treatment with CD increased hepatic inflammation and mild elevations in fibrosis in our experimental model.

### Treatment With 2-Hydroxypropyl-β-cyclodextrin Normalizes Lysosomal Size of Metabolically Challenged Bone Marrow-Derived Macrophages

**Figure 3:**
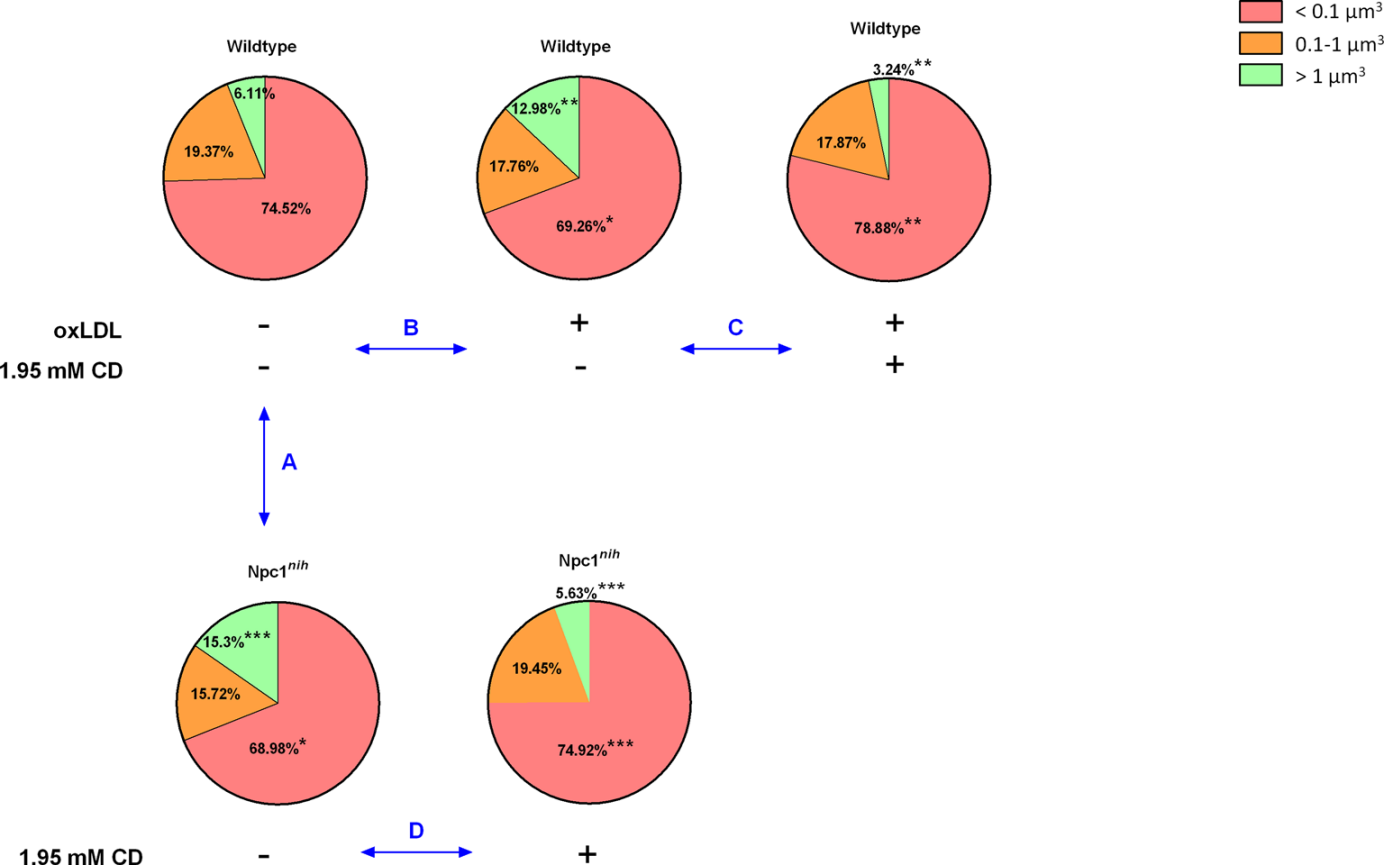
Lysosomal volume of metabolically-challenged bone marrow-derived macrophages with and without intervention. We quantified the number of lysosomes, categorized them by size (< 0.1 µm^3^; 0.1-1 µm^3^; > 1 µm^3^) and expressed the number of lysosomes per size relative to the total amount of lysosomes present inside the BMDM. ***Comparison A*:** Relative comparison of the distribution of lysosomal volumes between *Wt* and *Npc1^nih^* BMDM. ***Comparison B*** *:* Relative comparison of the distribution of lysosomal volumes between control and oxLDL (25µg/ml; 72hr) -stimulated *Wt* BMDM. ***Comparison C***: Relative comparison of the distribution of lysosomal volumes between oxLDL-stimulated *Wt* BMDM treated with or without CD (1.95 mM; 4hr). ***Comparison D*** *:* Relative comparison of the distribution of lysosomal volumes between *Npc1^nih^* BMDM treated with or without CD. ^*^, ^**^ and ^***^ indicate *p* < 0.05, 0.01 and 0.001 resp.by means of unpaired t-test. Data are the result of analysis of n = 5-7 BMDM per experimental condition.

As CD treatment mobilized cholesterol, but increased inflammation in our *in vivo* model for lysosomal cholesterol-induced inflammation, we opted to investigate the effect of CD on lysosomal size in metabolically challenged BMDM *via* confocal microscopy. For this purpose, we first compared lysosomal size between wildtype and *Npc1^nih^* BMDM. While *Npc1^nih^* BMDM showed a reduced number of small lysosomes (categorized as < 0.1 µm^3^), they showed an increase in the number of large lysosomes (categorized as > 1 µm^3^) as compared to wildtype BMDM, which is likely the result of lysosomal lipid accumulation that is characteristic for the mutation (**Figure 3 Comparison A**; **Supplementary Table 2**).

As internalization of oxidized cholesterol-rich low-density lipoprotein (oxLDL) in BMDM also leads to lysosomal accumulation, we next compared lysosomal size of oxLDL (25µg/ml; 72hr)-incubated wildtype BMDM to control-incubated wildtype BMDM. Incubating wildtype BMDM with oxLDL similarly reduced the number of small lysosomes and increased the number of large lysosomes, confirming the effect of oxLDL on lysosomal size (**Figure 3 Comparison B**; **Supplementary Table 3**) as well as our method to quantify the number of lysosomes based on their size. Representative videos of all indicated conditions are shown in **Supplementary Videos 1**–**5**.

Next, to assess the impact of CD treatment, we compared lysosomal size of oxLDL-incubated wildtype BMDM that were treated with or without CD (1.95 mM; 4hr). As expected, CD treatment increased the number of small lysosomes and reduced the level of large lysosomes, thereby reversing the oxLDL-induced effects on lysosomal size (**Figure 3 Comparison C**; **Supplementary Table 4**). Furthermore, similar findings were observed when *Npc1^nih^* BMDM were treated with CD (**Figure 3 Comparison D**; **Supplementary Table 5**). Overall, these findings confirm the beneficial effect of CD treatment on lysosomes of metabolically challenged BMDM.

### Incubation With 2-Hydroxypropyl-β-cyclodextrin Exerts Pro-Inflammatory Effects in Metabolically Challenged Bone Marrow Derived Macrophages

**Figure 4:**
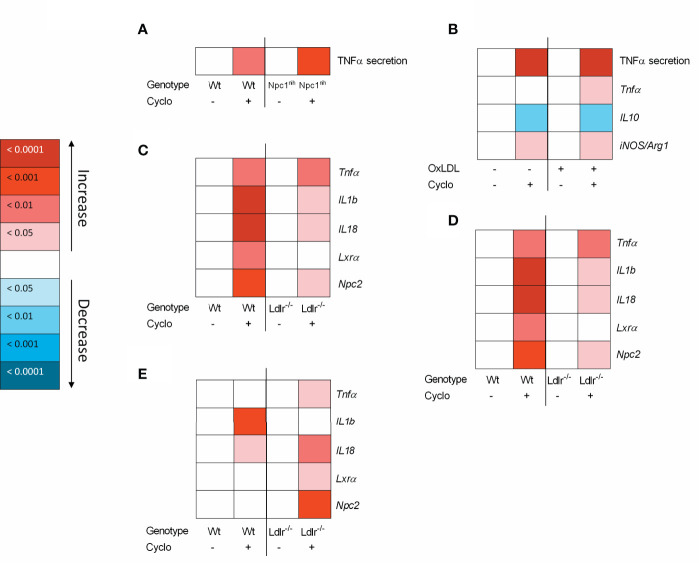
Metabolically-challenged BMDM parameters of inflammation and cholesterol metabolism after four hour incubation with 2-hydroxypropyl-β-cyclodextrin. **(A)** TNFα protein levels of 2-hydroxypropyl-β-cyclodextrin (1.95 mM; 4hr)-treated *Wt* or *Npc1^nih^* BMDM that were terminally stimulated with LPS (100ng/ml; 4hr). **(B)** TNFα protein levels and *Tnfα, IL10* and *iNOS/Arg1* ratio gene expression data of oxLDL (25µg/ml; 24hr)-exposed *Npc1^nih^* BMDM treated with or without CD (4hr) that were terminally stimulated with LPS (100ng/ml; 4hr). Gene expression data were set relative to control-exposed *Npc1^nih^* BMDM treated with saline and stimulated with LPS. **(C–E)** Gene expression analysis of *Tnfα, IL-1b, IL-18, Lxrα* and *Npc2* of oxLDL (25µg/ml; 24hr)-exposed *Ldlr^-/-^* and *Wt* BMDM treated with or without CD (1.95 mM; 4hr) and terminally stimulated with control **(C)**, Pam3Cys (4hr) **(D)** or LPS (4hr) **(E)**. Gene expression data were set relative to oxLDL-exposed Wt BMDM treated with saline. Colored (red or blue) boxes are compared to the box directly at their left *via* two-way ANOVA followed by Tukey post-hoc analysis, indicating the effect of CD. Data are the result of 2 or 3 independent experiments.

To increase our insight into the inflammatory effects of CD, we performed a series of *in vitro* experiments in BMDM, framed within the context of metabolic inflammation. First, we incubated wildtype and *Npc1^nih^* BMDM with CD for 4hr followed by 4hr lipopolysaccharide (LPS) stimulation. Four-hour CD treatment increased TNFα protein secretion in both wildtype and *Npc1^nih^* BMDM, suggesting an acute pro-inflammatory effect of CD also in wildtype and *Npc1^nih^* BMDM (**Figure 4A**; **Supplementary Figure S5A**). To provide stronger evidence for this inflammatory effect of CD, *Npc1^nih^* BMDM were challenged with oxLDL, followed by 4hr CD incubation and 4hr LPS stimulation, thereby creating a more severe model of metabolic inflammation. Similar to our previous findings, CD treatment increased TNFα protein secretion in both oxLDL- and control-challenged *Npc1^nih^* BMDM (**Figure 4B**; **Supplementary Figure S5B**). This was confirmed at gene expression level, showing increased levels of pro-inflammatory markers (*Tnfα*, *iNos/Arg1* ratio) (**Figure 4B**; **Supplementary Figure S5B**) and decreased levels of anti-inflammatory markers (*IL-10*; **Figure 4B**; **Supplementary Figure S5B**) after CD treatment. These pro-inflammatory findings of CD were also confirmed in a separate BMDM experiment with similar set-up in wildtype BMDM (**Supplementary Figure S6A**) and without the final LPS stimulus (**Supplementary Figure S6B**).

As another model for metabolic disturbance, we investigated the inflammatory effects of CD on *Ldlr* ^-/-^ BMDM, which is a mutation causing disturbances in cholesterol metabolism. Twenty-four-hour incubation with oxLDL followed by 4hr CD treatment resulted in increased gene expression levels of *Tnfα, IL-1b*, and *IL-18*, confirming the pro-inflammatory effects of CD also in *Ldlr^-/-^* BMDM (**Figure 4C**; **Supplementary Figure S4C**). Nevertheless, CD treatment increased *Lxrα* and *Npc2* expression, confirming the cholesterol-mobilizing properties of CD. To further confirm these findings, a similar experiment was conducted with the addition of a final stimulation with Pam3Cys or LPS for 4hr. Stimulation with Pam3Cys (**Figure 4D**; **Supplementary Figure S4D**) and LPS (**Figure 4E**; **Supplementary Figure S4E**) showed similar results as previously described, showing pro-inflammatory and cholesterol-mobilizing effects of CD treatment in *Ldlr^-/-^* BMDM. Together, these findings show that while CD maintains its effect on cholesterol metabolism, pro-inflammatory effects become apparent after 4hr incubation in metabolically challenged BMDM.

### Incubation With 2-Hydroxypropyl-β-cyclodextrin Exerts Pro-Inflammatory Effects in Macrophage-Derived Cell Lines

**Figure 5:**
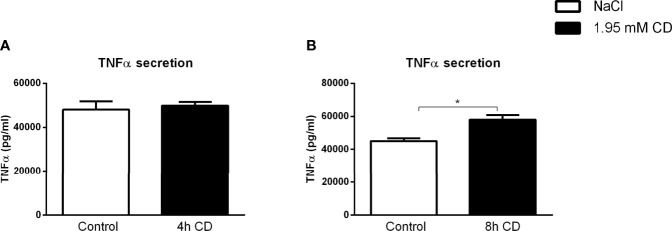
RAW cell line-related parameters of inflammation after prolonged exposure to 2-hydroxypropyl-β-cyclodextrin. **(A, B)** TNFα protein levels of 2-hydroxypropyl-β-cyclodextrin (1.95 mM; 4 or 8hr)-treated RAW cells that were terminally stimulated with LPS (100ng/ml; 4hr). Data represent n = 3 for each experimental group. * indicates p < 0.05 by means of unpaired t-test.

**Figure 6:**
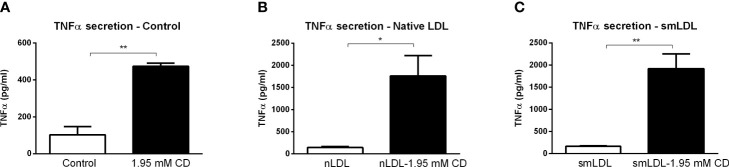
THP1 cell line-related parameters of inflammation after prolonged exposure to 2-hydroxypropyl-β-cyclodextrin. **(A–C)** TNFα protein levels of control, nLDL or smLDL (24hr)-exposed THP1 cells treated with or without 2-hydroxypropyl-β-cyclodextrin (1.95 mM; 4hr). ^*^, ^**^ indicates *p* < 0.05 and 0.01 by means unpaired t-test. Data represent n = 3 for each experimental group.

To validate the inflammatory effect of CD in non-primary cells, we investigated the impact of CD treatment on the macrophage-derived mouse RAW 264.7 and human THP-1 cell lines. Firstly, RAW 264.7 cells were incubated with CD for 4 or 8hr, followed by 4hr LPS stimulation. While 4hr incubation did not show any inflammatory effect, CD treatment for 8hr increased TNFα protein secretion (**Figures 5A, B**; **Supplementary Figures S7A, B**). Furthermore, THP-1-derived macrophages were challenged with sphingomyelinase-aggregated LDL (smLDL), native LDL or control for 24hr and incubated with CD for 4hr. Secreted protein levels of TNFα increased in all three experimental groups after CD incubation (**Figures 6A–C**). Together, these results indicate that CD also shows pro-inflammatory properties in macrophage-derived cell lines.

### The Pro-Inflammatory Effect of 2-Hydroxypropyl-β-cyclodextrin on Bone Marrow-Derived Macrophages Is Time- and Concentration-Dependent

**Figure 7:**
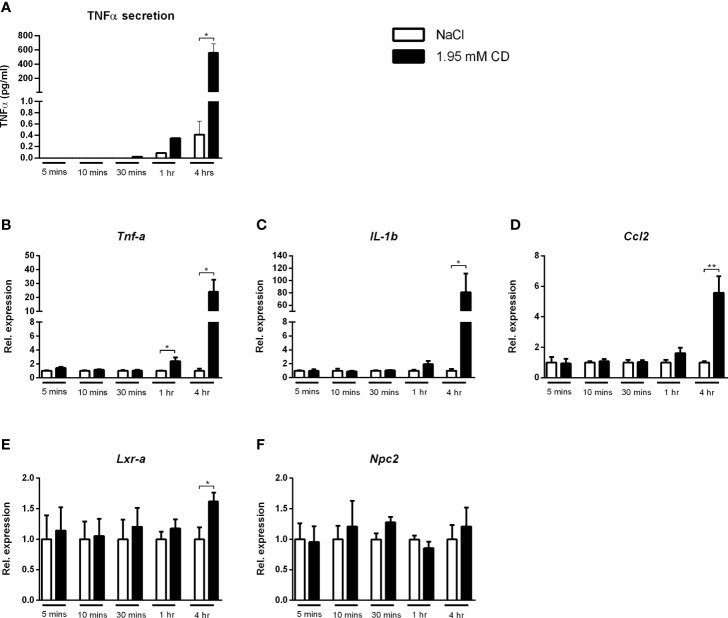
Time dependency of the inflammatory effect of 2-hydroxypropyl-β-cyclodextrin. **(A)** TNFα protein levels of *Wt* BMDM treated with 2-hydroxypropyl-β-cyclodextrin for different time durations. **(B–D)** Gene expression analysis of *Tnfα, IL-1b, Ccl2.* **(E, F)** Gene expression analysis of *Lxrα and Npc2.* Gene expression data were set relative to *Wt* BMDM treated with saline. Data are the result of 2 independent experiments. ^*^, ^**^ indicates *p* < 0.05 and 0.01 by means of unpaired t-test.

As increased depletion of cholesterol from the plasma membrane was shown to result in pro-inflammatory responses (31), we first investigated whether incubation time influenced the pro-inflammatory effect of CD. For this purpose, wildtype BMDMs were incubated with CD for 5, 10 and 30 min as well as for 1 and 4hr. Protein secretion of TNFα showed a marked time-dependent response after CD treatment (**Figure 7A**). This time-dependent, pro-inflammatory effect of CD was confirmed at gene expression levels for the inflammatory markers *Tnfα*, *IL-1b* and *Ccl2*, only showing increased expression after 1 and 4hr incubation (**Figures 7B–D**). *Lxrα* showed a similar increase after 4 hours of CD incubation, *Npc2* only showed a trend (**Figures 7E, F**). Expression levels of *IL-18*, *Arg1, Abca1, Abcg1* and *Npc1* remained similar (**Supplementary Figure S8**).

**Figure 8:**
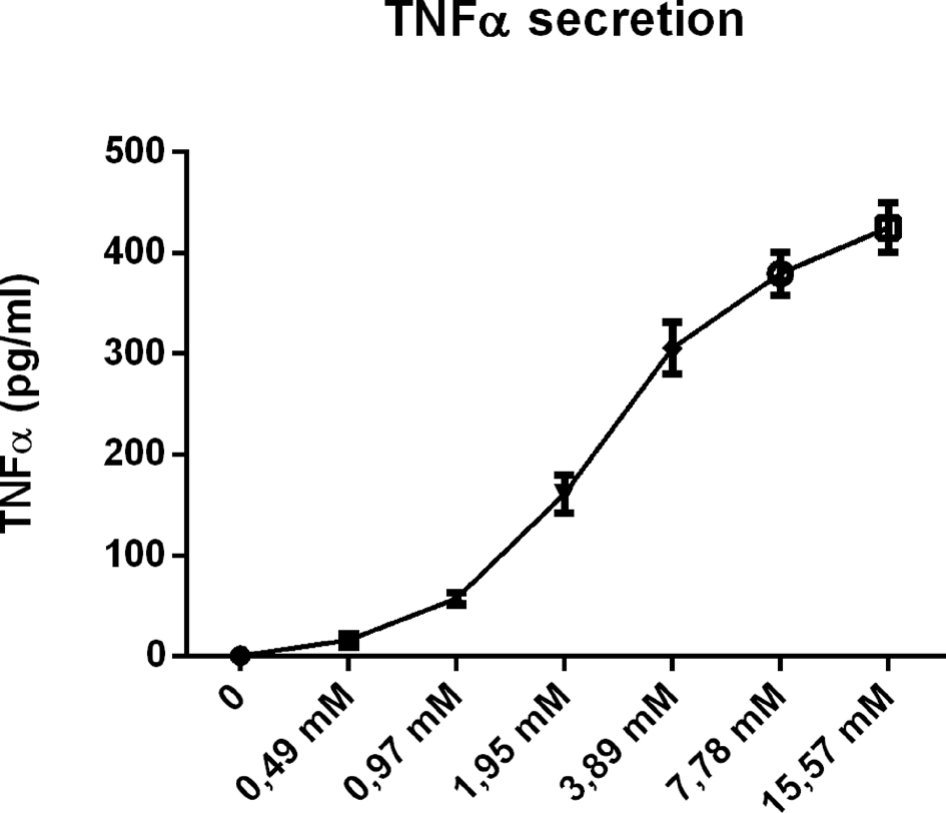
Concentration dependency of the inflammatory effect of 2-hydroxypropyl-β-cyclodextrin. Dose response analysis of TNFα protein levels of *Wt* BMDM treated with 2-hydroxypropyl-β-cyclodextrin for 4hrs.

To further confirm our hypothesis, we also performed a dose-response curve with CD (4hr incubation) in wildtype BMDMs. As indicated in **Figure 8**, CD incubation showed a clear concentration-dependent effect on TNFα secretion, adding fuel to our argument that the pro-inflammatory effects of CD are related to cholesterol depletion from the plasma membrane.

## Discussion

New perspectives to reduce cholesterol levels aim at improving cellular instead of plasma cholesterol levels, raising the argument to use CD as a pharmacological compound to improve cholesterol homeostasis. Here, we confirm the cholesterol-depleting effects of CD in a metabolic inflammatory context, but concomitantly show a detrimental time- and concentration- dependent inflammatory effect of CD treatment. Therefore, while CD is able to decrease cellular cholesterol levels, our findings demonstrate that its use in the clinic should be closely monitored especially in patients with a metabolic inflammatory background.

Our observation that CD treatment promotes cellular cholesterol mobilization, but induces a time- and concentration-dependent inflammatory effect implies the importance of the subcellular distribution of cholesterol and the subsequent impact of cholesterol depletion from these specific locations. Unesterified cholesterol serves a key structural function in the plasma membrane as it is critical for the formation of liquid-ordered rafts, which determine membrane fluidity. The membrane fluidity is therefore coupled to the free cholesterol/phospholipid ratio of the plasma membrane as this ratio determines the formation of the aforementioned rafts (32, 33). Under hyperlipidemic conditions, the plasma membrane serves as the first pool to deposit free cholesterol from external sources (34). However, accumulation of free cholesterol in the plasma membrane above an optimal free cholesterol/phospholipid ratio negatively impacts plasma membrane fluidity (33). Indeed, Yvan-Charvet et al. showed that increased accumulation of free cholesterol in the plasma membrane (induced by *Abca1* or *Abcg1* knockout) directly results in pro-inflammatory responses, supporting our current notion of the limited physiological capacity of the plasma membrane to harbor free cholesterol (31). Therefore, exceeding the plasma membrane’s capacity to harbor cholesterol directly results in intracellular cholesterol accumulation (mainly in lysosomes), which is considered a severe pathological phenomenon for mediating inflammatory responses (4). Evidently, under conditions that generate severe accumulation of intracellular cholesterol (exemplified by the NPC1 mutation and LDLR knockout), CD-mediated depletion of cholesterol is highly desirable. In line with this concept, the increased lysosomal size in metabolically challenged macrophages was reverted after incubation with CD treatment. This advantageous cholesterol-depleting effect of CD has been confirmed in multiple previous reports (15, 18, 31, 35). In contrast, in the current study, we consistently show a harmful, pro-inflammatory (and even mild pro-fibrotic) effect of CD under cholesterol-induced inflammatory conditions *in vivo* and *in vitro*. This pro-inflammatory effect can be rationalized by the prolonged cellular exposure to CD, which influences plasma membrane cholesterol combined with intracellular cholesterol levels. Indeed, given the key role of cholesterol in the formation of liquid-ordered rafts, excessive depletion of plasma membrane cholesterol (as is induced with CD treatment) is highly undesirable as it disrupts these rafts, affecting membrane fluidity. This rationale is further supported by findings showing that cholesterol extraction of the plasma membrane is a primary location where CD exerts its function (36, 37). Therefore, excessive depletion of cholesterol from the plasma membrane by CD might be an explanation for the observed pro-inflammatory findings in our study as well as the time-and concentration-dependent character of our findings. The time-dependent findings are also in line with experiments reported by Pilely *et al*. and Ding *et al*., that show that while incubation of different types of cyclodextrin for a short period of time (10 and 30 min) is anti-inflammatory (35), incubation for 24 and 48hr lead to ototoxicity (19). Together, these observations indicate that while CD has potential in advantageously depleting cellular cholesterol, it is essential to monitor the quantity of cellular cholesterol and adjust the therapeutic dose/time accordingly to prevent undesired pro-inflammatory side-effects.

Notably, while neutrophils generally constitute the major immune cell population under inflammatory conditions, in the current study we only focused on the effect of CD on macrophages. This choice is based on previous findings by our group that demonstrate macrophages to be the most important immune cell in the *in vivo* model here described (as evidenced by changes in hematopoiesis (38) and organ-specific inflammation (22)). Nevertheless, as neutrophil aberrations have also been described in NPC1 disease (39), future research should further explore the involvement of neutrophils in CD-induced inflammatory responses in a metabolic inflammatory context.

These dichotomic characteristics of CD raise the question whether structural manipulation of cyclodextrins can reduce the harmful pro-inflammatory effects, while maintaining the advantageous cholesterol-depleting effects of CD. Cyclodextrins are composed of cyclic oligosaccharides of 6, 7 or 8 glucose units (referred to as α-, β- and γ-cyclodextrins respectively), providing cyclodextrin a polar and hydrophilic surface combined with a non-polar cavity (40). This dual-property structure of CD grants itself for modification *via* polymerization, creating so-called polycyclodextrins. Indeed, various modifications have been performed on cyclodextrin, creating more efficient and effective cyclodextrins for a plethora of purposes (40). In line, Kulkarni et al. adapted the structure CD (the type we employed in our study) and showed that their linear degradable, high molecular weight polymer variation improved the pharmacokinetic profile and bioavailability in NPC mice (41). Moreover, using polyrotaxanes enabled specific release of CD inside lysosomes, thereby minimizing the effect on plasma membrane cholesterol (42). Based on these reports, it is anticipated that designing polycyclodextrins is a promising approach that can have a considerable clinical impact due to its ability to reduce the pro-inflammatory properties of CD described in our study.

In conclusion, though we confirm its cholesterol-depleting effects, we here demonstrate a time- and concentration-dependent harmful pro-inflammatory effect of CD under metabolic inflammatory conditions. As such, we suggest that clinical use of CD, in particular in a metabolic inflammatory context, should be closely monitored to prevent undesired side effects.

## Data Availability Statement

The raw data supporting the conclusions of this article will be made available by the authors, without undue reservation.

## Ethics Statement

The animal study was reviewed and approved by Animal Experiment Committee of Maastricht University.

## Author Contributions

TH, TY, and RS-S conceived the study and designed the experiments. TH, TY, MG, JL, DK, MZ, MW, JP, DiL, and DaL contributed to sample collection, data collection, molecular experiments, and data analysis. TH, TY, and RS-S wrote the manuscript. All authors helped in editing and revising the manuscript. RS-S obtained funding. All authors contributed to the article and approved the submitted version.

## Funding

This work was supported by the TKI-LSH (grant no. 40-41200-98-9306). MW was supported by VIDI grant 917.15.350 and an Aspasia grant from the Netherlands Organization of Scientific Research (NWO) and a Rosalind Franklin Fellowship from the University of Groningen.

## Conflict of Interest

The authors declare that the research was conducted in the absence of any commercial or financial relationships that could be construed as a potential conflict of interest.

## Publisher’s Note

All claims expressed in this article are solely those of the authors and do not necessarily represent those of their affiliated organizations, or those of the publisher, the editors and the reviewers. Any product that may be evaluated in this article, or claim that may be made by its manufacturer, is not guaranteed or endorsed by the publisher.

## Supplementary Material

Supplementary Figure 1 Overview of experimental set-up of *in vivo* experiment.

Supplementary Figure 2 Relative organ weights.

Supplementary Figure 3 Quantification of hepatic HE staining of *Npc1^nih^*-tp mice.

Supplementary Figure 4 Hepatic gene expression of fibrotic markers.

Supplementary Figure 5 Experimental set-ups related to **Figure 4**.

Supplementary Figure 6 Heatmap of inflammatory mediators of CD-treated Wt BMDM. **(A, B)** TNFα protein levels and *Tnfα, IL1b, IL-10, Ccl2, Lxrα* and *Npc2* gene expression of oxLDL (24hr)-exposed Wt BMDM treated with or without CD (4hr) that were terminally stimulated with LPS **(A)** or without **(B)** LPS for 4 hr. Gene expression data were set relative to control-exposed *Wt* BMDM treated with saline and stimulated with **(A)** or without **(B)** LPS. Colored (red or blue) boxes are compared to the box directly at their left *via* two-way ANOVA followed by Tukey post-hoc analysis, indicating the effect of CD. Data are the result of 2-4 independent experiments.

Supplementary Figure 7 Experimental set-ups related to **Figure 5**.

Supplementary Figure 8 Gene expression analysis of BMDM exposed to different time intervals of CD.

Supplementary Table 1 Primer sequences of genes used for quantitative RT-PCR.

Supplementary Table 2 Raw data related to confocal quantification of conditions Wt control vs. NPC1 control. Blue numbers are used for statistical analysis.

Supplementary Table 3 Raw data related to confocal quantification of conditions Wt control vs. Wt oxLDL. Blue numbers are used for statistical analysis.

Supplementary Table 4 Raw data related to confocal quantification of conditions Wt-oxLDL-control vs. Wt-oxLDL-CD. Blue numbers are used for statistical analysis.

Supplementary Table 5 Raw data related to confocal quantification of conditions NPC1 control vs. NPC1 CD. Blue numbers are used for statistical analysis.

Supplementary Video 1 wildtype control.

Supplementary Video 2 Npc1^nih^ control.

Supplementary Video 3 wildtype oxLDL.

Supplementary Video 4 wildtype oxLDL CD.

Supplementary Video 5 Npc1^nih^ CD.
