# Supplementary figures and images for: Pro-Inflammatory Implications of 2-Hydroxypropyl-β-cyclodextrin Treatment
Source: Front Immunol. 2021 Aug 20;12:716357. doi: 10.3389/fimmu.2021.716357 (PMC8417873; doi:10.3389/fimmu.2021.716357)

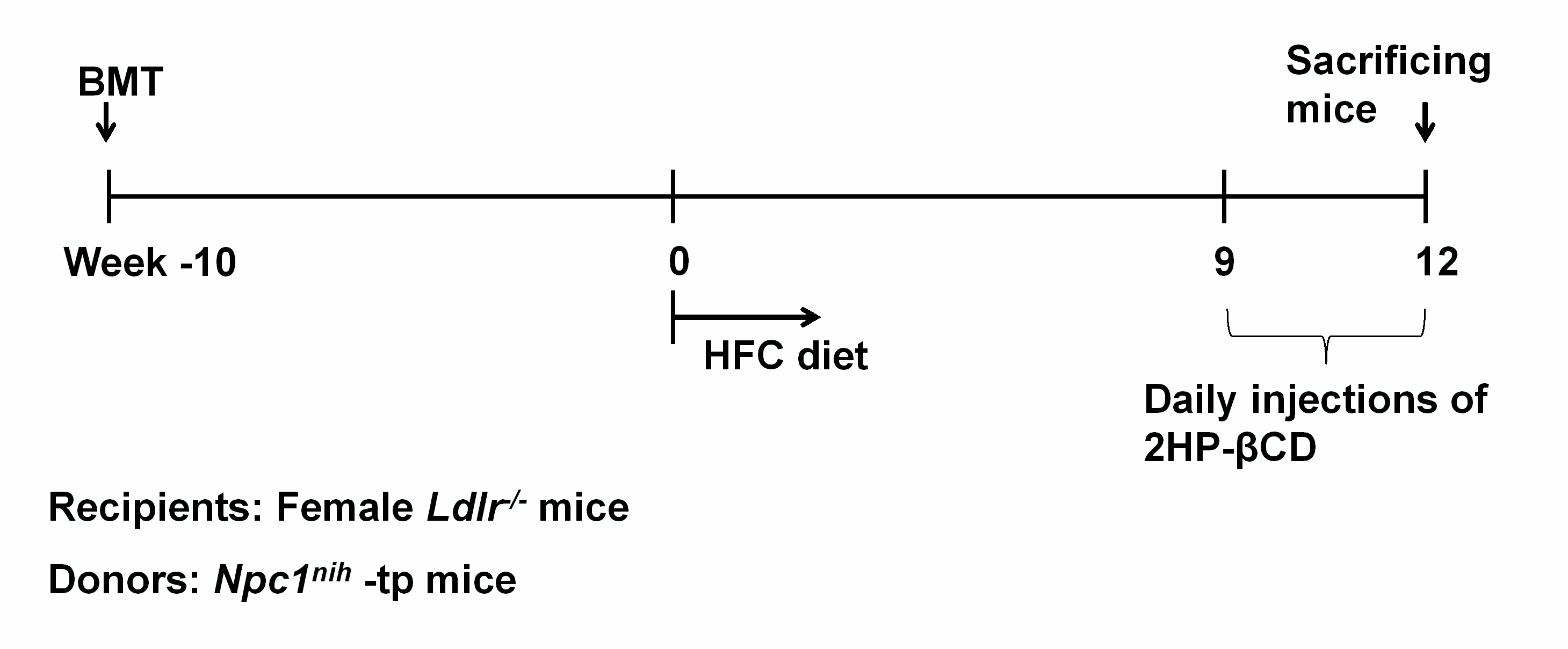

Supplement: Supplementary Figure 1 — Overview of experimental set-up of in vivo experiment. [file Image_1.tif]

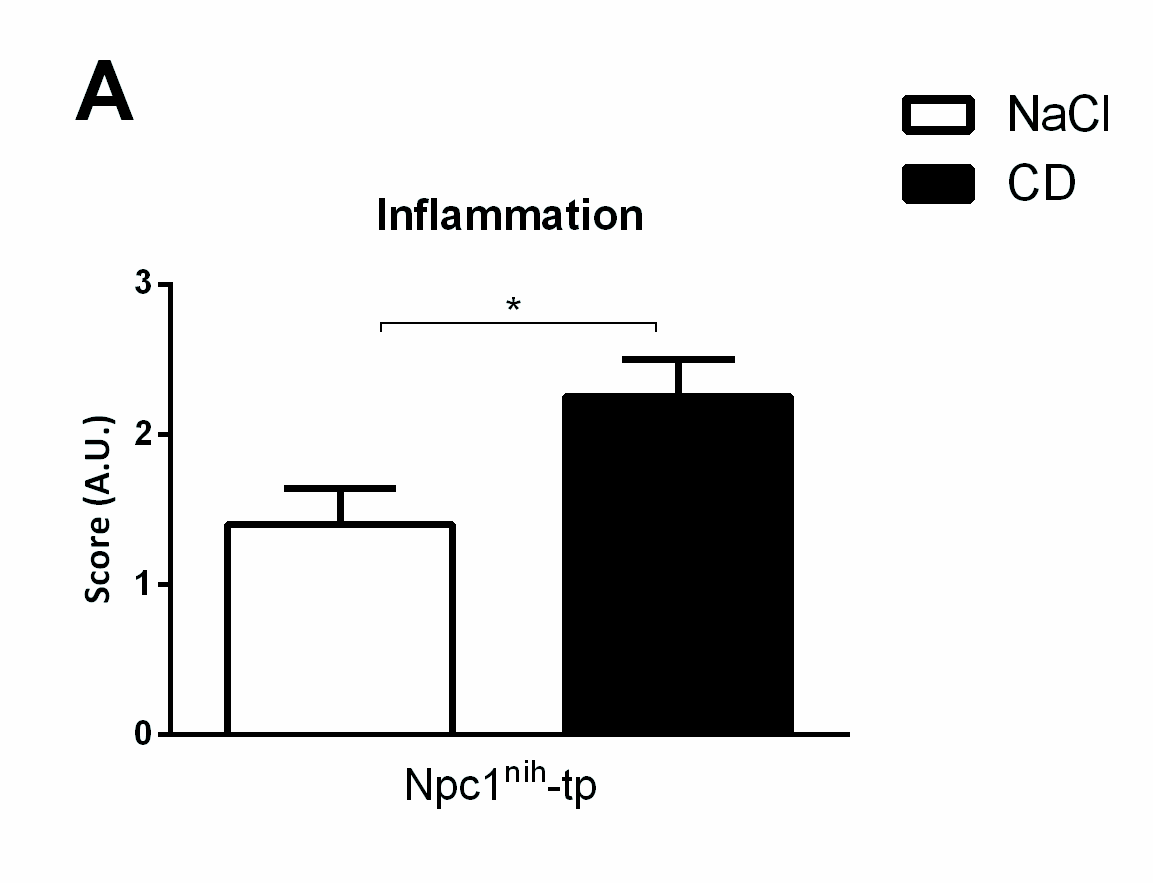

Supplement: Supplementary Figure 3 — Quantification of hepatic HE staining of Npc1nih-tp mice. [file Image_3.tif]

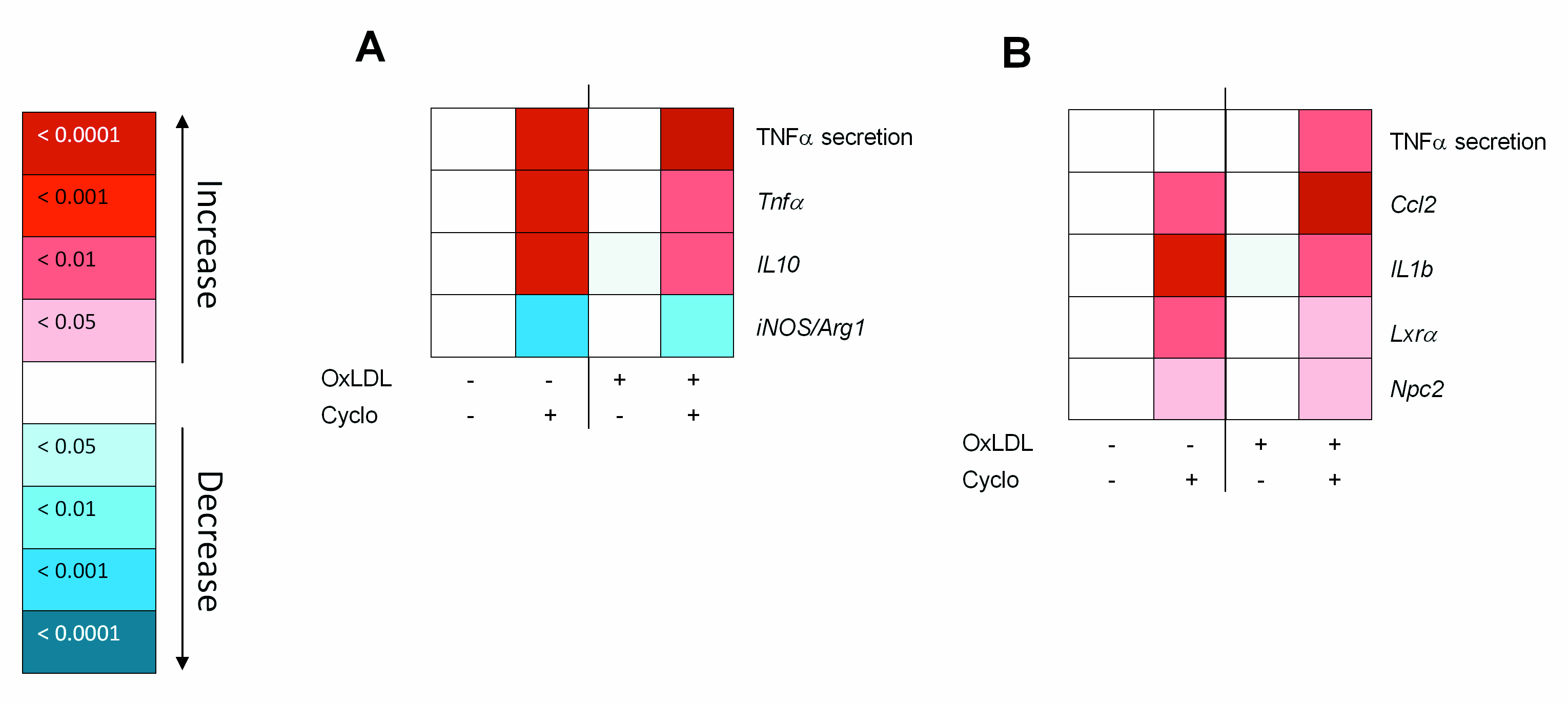

Supplement: Supplementary Figure 6 — Heatmap of inflammatory mediators of CD-treated Wt BMDM. (A, B) TNFα protein levels and Tnfα, IL1b, IL-10, Ccl2, Lxrα and Npc2 gene expression of oxLDL (24hr)-exposed Wt BMDM treated with or without CD (4hr) that were terminally stimulated with LPS (A) or without (B) LPS for 4 hr. Gene expression data were set relative to control-exposed Wt BMDM treated with saline and stimulated with (A) or without (B) LPS. Colored (red or blue) boxes are compared to the box directly at their left via two-way ANOVA followed by Tukey post-hoc analysis, indicating the effect of CD. Data are the result of 2-4 independent experiments. [file Image_6.tif]
