# Supplementary material for: Pro-Inflammatory Implications of 2-Hydroxypropyl-β-cyclodextrin Treatment
Source: Front Immunol. 2021 Aug 20;12:716357. doi: 10.3389/fimmu.2021.716357 (PMC8417873; doi:10.3389/fimmu.2021.716357)
Supplement: Supplementary Table 1 — Primer sequences of genes used for quantitative RT-PCR. [file Table_1.docx]

| **Gene name** | **Forward primer 5’-3’** | **Reverse primer 5’-3’** |
| --- | --- | --- |
| Cyp7a1 | CATTACAGAGTGCTGGCCAAGA | CGCAGAGCCTCCTTGATGAT |
| Cyp27a1 | CTGCACTTCCTGCTGACCAAT | AGGGCCCATGTCAGTGTGTT |
| Tnfα | CATCTTCTCAAAATTCGAGTGACAA | TGGGAGTAGACAAGGTACAACCC |
| Ccl2 | GCTGGAGAGCTACAAGAGGATCA | TCTCTCTTGAGCTTGGTGACAAAA |
| Ccl5 | GGAGTATTTCTACACCAGCAGCAA | GCGGTTCCTTCGAGTGACA |
| Saa1 | GGCTGCTGAGAAAATCAGTGATG | TCAGCAATGGTGTCCTCATGTC |
| iNOS | GCAAACCCAAGGTCTACGTTCA | CCTCATTGGCCAGCTGCTT |
| Arg1 | CATGGGCAACCTGTGTCCTT | CGATGTCTTTGGCAGATATGCA |
| IL-10 | GCTCTTACTGACTGGCATGAG | CGCAGCTCTAGGAGCATGTG |
| IL-1B | AAAGAATCTATACCTGTCCTGTGTAATGAAA | GGTATTGCTTGGGATCCACACT |
| IL-18 | GACTCTTGCGTCAACTTCAAGG | CAGGCTGTCTTTTGTCAACGA |
| Lxr-a | CAACAGTGTAACAGGCGCT | TGCAATGGGCCAAGGC |
| Npc2 | CGGAGCCCCTGCACTTC | ACAGGGATCGGTGGGACAT |
| Abca1 | CCCAGAGCAAAAAGCGACTC | GGTCATCATCACTTTGGTCCTTG |
| Abcg1 | TCGGACGCTGTGCGTTTT | CCCACAAATGTCGCAACCT |
| Npc1 | ATATAACGAGAGCATTCACCATGAGTA | TAACACCACGATCCCTCCAAA |
| Cyclophilin  (housekeeping) | TTCCTCCTTTCACAGAATTATTCCA | CCGCCAGTGCCATTATGG |
| Hprt  (housekeeping) | TCAGTCAACGGGGGACATAAA | GGGGCTGTACTGCTTAACCAG |

**Supplementary Table 1:** **Primer sequences of genes used for quantitative RT-PCR**
