# Supplementary material for: Pro-Inflammatory Implications of 2-Hydroxypropyl-β-cyclodextrin Treatment
Source: Front Immunol. 2021 Aug 20;12:716357. doi: 10.3389/fimmu.2021.716357 (PMC8417873; doi:10.3389/fimmu.2021.716357)
Supplement: Supplementary Table 2 — Raw data related to confocal quantification of conditions Wt control vs. NPC1 control. Blue numbers are used for statistical analysis. [file Table_2.docx]

| *WT control* |  | | |  |  |  |  | *NPC1 control* |  |  |  |  |  |  |  |
| --- | --- | --- | --- | --- | --- | --- | --- | --- | --- | --- | --- | --- | --- | --- | --- |
|  | *Absolute numbers* | | |  | *Fractions (%)* | | |  | *Absolute numbers* | | |  | *Fractions (%)* | | |
|  | <0.1 | 0.1-1 | >1 | ***Sum*** | <0.1 | 0.1-1 | >1 |  | <0.1 | 0.1-1 | >1 | ***Sum*** | <0.1 | 0.1-1 | >1 |
| ***Bmdm 1*** | 231 | 46 | 9 | *286* | 80,77 | 16,08 | 3,15 | ***Bmdm 1*** | 157 | 28 | 40 | *225* | 69,78 | 12,44 | 17,78 |
| ***Bmdm 2*** | 175 | 30 | 16 | *221* | 79,19 | 13,57 | 7,24 | ***Bmdm 2*** | 77 | 13 | 22 | *112* | 68,75 | 11,61 | 19,64 |
| ***Bmdm 3*** | 137 | 49 | 18 | *204* | 67,16 | 24,02 | 8,82 | ***Bmdm 3*** | 378 | 113 | 66 | *557* | 67,86 | 20,29 | 11,85 |
| ***Bmdm 4*** | 187 | 56 | 12 | *255* | 73,33 | 21,96 | 4,71 | ***Bmdm 4*** | 505 | 152 | 90 | *747* | 67,60 | 20,35 | 12,05 |
| ***Bmdm 5*** | 222 | 55 | 14 | *291* | 76,29 | 18,90 | 4,81 | ***Bmdm 5*** | 237 | 61 | 46 | *344* | 68,90 | 17,73 | 13,37 |
| ***Bmdm 6*** | 140 | 41 | 19 | *200* | 70,00 | 20,50 | 9,50 | ***Bmdm 6*** | 137 | 23 | 33 | *193* | 70,98 | 11,92 | 17,10 |
| ***Bmdm 7*** | 346 | 95 | 21 | *462* | 74,89 | 20,56 | 4,55 |  |  |  |  |  |  |  |  |
| ***Average*** | 1438 | 372 | 109 | *1919* | **74,52** | **19,37** | **6,11** |  | 1491 | 390 | 297 | *2178* | **68,98** | **15,72** | **15,30** |

**Supplementary Table 2**
