# Supplementary material for: Pro-Inflammatory Implications of 2-Hydroxypropyl-β-cyclodextrin Treatment
Source: Front Immunol. 2021 Aug 20;12:716357. doi: 10.3389/fimmu.2021.716357 (PMC8417873; doi:10.3389/fimmu.2021.716357)
Supplement: Supplementary Table 3 — Raw data related to confocal quantification of conditions Wt control vs. Wt oxLDL. Blue numbers are used for statistical analysis. [file Table_3.docx]

**Supplementary Table 3**

| *WT control* |  | | |  |  |  |  | *WT oxLDL* |  |  |  |  |  |  |  |
| --- | --- | --- | --- | --- | --- | --- | --- | --- | --- | --- | --- | --- | --- | --- | --- |
|  | *Absolute numbers* | | |  | *Fractions (%)* | | |  | *Absolute numbers* | | |  | *Fractions (%)* | | |
|  | <0.1 | 0.1-1 | >1 | ***Sum*** | <0.1 | 0.1-1 | >1 |  | <0.1 | 0.1-1 | >1 | ***Sum*** | <0.1 | 0.1-1 | >1 |
| ***Bmdm 1*** | 231 | 46 | 9 | *286* | 80,77 | 16,08 | 3,15 | ***Bmdm 1*** | 207 | 53 | 31 | *291* | 71,13 | 18,21 | 10,65 |
| ***Bmdm 2*** | 175 | 30 | 16 | *221* | 79,19 | 13,57 | 7,24 | ***Bmdm 2*** | 161 | 52 | 33 | *246* | 65,45 | 21,14 | 13,41 |
| ***Bmdm 3*** | 137 | 49 | 18 | *204* | 67,16 | 24,02 | 8,82 | ***Bmdm 3*** | 209 | 42 | 61 | *312* | 66,99 | 13,46 | 19,55 |
| ***Bmdm 4*** | 187 | 56 | 12 | *255* | 73,33 | 21,96 | 4,71 | ***Bmdm 4*** | 311 | 88 | 47 | *446* | 69,73 | 19,73 | 10,54 |
| ***Bmdm 5*** | 222 | 55 | 14 | *291* | 76,29 | 18,90 | 4,81 | ***Bmdm 5*** | 835 | 186 | 123 | *1144* | 72,99 | 16,26 | 10,75 |
| ***Bmdm 6*** | 140 | 41 | 19 | *200* | 70,00 | 20,50 | 9,50 |  |  |  |  |  |  |  |  |
| ***Bmdm 7*** | 346 | 95 | 21 | *462* | 74,89 | 20,56 | 4,55 |  |  |  |  |  |  |  |  |
| ***Average*** | 1438 | 372 | 109 | *1919* | **74,52** | **19,37** | **6,11** |  | 1723 | 421 | 295 | *2439* | **69,26** | **17,76** | **12,98** |
