# Supplementary material for: Pro-Inflammatory Implications of 2-Hydroxypropyl-β-cyclodextrin Treatment
Source: Front Immunol. 2021 Aug 20;12:716357. doi: 10.3389/fimmu.2021.716357 (PMC8417873; doi:10.3389/fimmu.2021.716357)
Supplement: Supplementary Table 4 — Raw data related to confocal quantification of conditions Wt-oxLDL-control vs. Wt-oxLDL-CD. Blue numbers are used for statistical analysis. [file Table_4.docx]

**Supplementary Table 4**

| *WT oxLDL - control* |  | | |  |  |  |  | *WT oxLDL -CD* |  |  |  |  |  |  |  |
| --- | --- | --- | --- | --- | --- | --- | --- | --- | --- | --- | --- | --- | --- | --- | --- |
|  | *Absolute numbers* | | |  | *Fractions (%)* | | |  | *Absolute numbers* | | |  | *Fractions (%)* | | |
|  | <0.1 | 0.1-1 | >1 | ***Sum*** | <0.1 | 0.1-1 | >1 |  | <0.1 | 0.1-1 | >1 | ***Sum*** | <0.1 | 0.1-1 | >1 |
| ***Bmdm 1*** | 207 | 53 | 31 | *291* | 71,13 | 18,21 | 10,65 | ***Bmdm 1*** | 489 | 104 | 2 | *595* | 82,18 | 17,48 | 0,34 |
| ***Bmdm 2*** | 161 | 52 | 33 | *246* | 65,45 | 21,14 | 13,41 | ***Bmdm 2*** | 301 | 43 | 2 | *346* | 86,99 | 12,43 | 0,58 |
| ***Bmdm 3*** | 209 | 42 | 61 | *312* | 66,99 | 13,46 | 19,55 | ***Bmdm 3*** | 509 | 123 | 21 | *653* | 77,95 | 18,84 | 3,22 |
| ***Bmdm 4*** | 311 | 88 | 47 | *446* | 69,73 | 19,73 | 10,54 | ***Bmdm 4*** | 368 | 81 | 8 | *457* | 80,53 | 17,72 | 1,75 |
| ***Bmdm 5*** | 835 | 186 | 123 | *1144* | 72,99 | 16,26 | 10,75 | ***Bmdm 5*** | 271 | 76 | 15 | *362* | 74,86 | 20,99 | 4,14 |
|  |  |  |  |  |  |  |  | ***Bmdm 6*** | 383 | 107 | 51 | *541* | 70,79 | 19,78 | 9,43 |
|  |  |  |  |  |  |  |  |  |  |  |  |  |  |  |  |
| ***Average*** | 1723 | 421 | 295 | *2439* | **69,26** | **17,76** | **12,98** |  | 2321 | 534 | 99 | *2954* | **78,88** | **17,87** | **3,24** |
