# Supplementary material for: Pro-Inflammatory Implications of 2-Hydroxypropyl-β-cyclodextrin Treatment
Source: Front Immunol. 2021 Aug 20;12:716357. doi: 10.3389/fimmu.2021.716357 (PMC8417873; doi:10.3389/fimmu.2021.716357)
Supplement: Supplementary Table 5 — Raw data related to confocal quantification of conditions NPC1 control vs. NPC1 CD. Blue numbers are used for statistical analysis. [file Table_5.docx]

**Supplementary Table 5**

| *NPC1 - control* |  | | |  |  |  |  | *NPC1 -CD* |  |  |  |  |  |  |  |
| --- | --- | --- | --- | --- | --- | --- | --- | --- | --- | --- | --- | --- | --- | --- | --- |
|  | *Absolute numbers* | | |  | *Fractions (%)* | | |  | *Absolute numbers* | | |  | *Fractions (%)* | | |
|  | <0.1 | 0.1-1 | >1 | ***Sum*** | <0.1 | 0.1-1 | >1 |  | <0.1 | 0.1-1 | >1 | ***Sum*** | <0.1 | 0.1-1 | >1 |
| ***Bmdm 1*** | 157 | 28 | 40 | *225* | 69,78 | 12,44 | 17,78 | ***Bmdm 1*** | 157 | 51 | 7 | *215* | 73,02 | 23,72 | 3,26 |
| ***Bmdm 2*** | 77 | 13 | 22 | *112* | 68,75 | 11,61 | 19,64 | ***Bmdm 2*** | 428 | 103 | 23 | *554* | 77,26 | 18,59 | 4,15 |
| ***Bmdm 3*** | 378 | 113 | 66 | *557* | 67,86 | 20,29 | 11,85 | ***Bmdm 3*** | 205 | 54 | 11 | *270* | 75,93 | 20,00 | 4,07 |
| ***Bmdm 4*** | 505 | 152 | 90 | *747* | 67,60 | 20,35 | 12,05 | ***Bmdm 4*** | 208 | 58 | 9 | *275* | 75,64 | 21,09 | 3,27 |
| ***Bmdm 5*** | 237 | 61 | 46 | *344* | 68,90 | 17,73 | 13,37 | ***Bmdm 5*** | 248 | 66 | 34 | *348* | 71,26 | 18,97 | 9,77 |
| ***Bmdm 6*** | 137 | 23 | 33 | *193* | 70,98 | 11,92 | 17,10 | ***Bmdm 6*** | 149 | 28 | 18 | *195* | 76,41 | 14,36 | 9,23 |
|  |  |  |  |  |  |  |  |  |  |  |  |  |  |  |  |
| ***Average*** | 1491 | 390 | 297 | *2178* | **68,98** | **15,72** | **15,30** |  | 1395 | 360 | 102 | *1857* | **74,92** | **19,45** | **5,63** |
