# Supplementary material for: Pro-Inflammatory Implications of 2-Hydroxypropyl-β-cyclodextrin Treatment
Source: Front Immunol. 2021 Aug 20;12:716357. doi: 10.3389/fimmu.2021.716357 (PMC8417873; doi:10.3389/fimmu.2021.716357)
Supplement: Supplementary file 19 [file DataSheet_1.docx]

**Supplementary methods**

**Macro created to quantify volumes and coordinates of lysosomes**

function isTIFF(filename) {

extensions=newArray("tif","tiff");

result=false;

for(i=0;i<extensions.length;i++) {

if(endsWith(toLowerCase(filename),"." + extensions[i]))

result = true;

}

return result;

}

setBatchMode(true);

inputdir=getDirectory("Choose directory containing TIFF images.");

imagefiles=getFileList(inputdir);

outputdir=getDirectory("Choose directory to save results.");

distance_between_centers=10 distance_max_contact=1.80");

for(i=0;i<imagefiles.length;i++) {

imagepath=inputdir + imagefiles[i];

imagename=replace(imagefiles[i],".tif","");

if(isTIFF(imagepath)) {

print("Processing file: " + imagepath + "...\n");

open(imagepath);

n=nSlices;

selectWindow(imagefiles[i]);

run("Properties...", "channels=1 slices=n frames=1 unit=micron pixel_width=0.169 pixel_height=0.169 voxel_depth=0.169 frame=[0 sec] origin=0,0");

run("Split Channels");

selectWindow(imagefiles[i] + " (red)");

run("Close");

NucleusChannel=imagefiles[i] + " (blue)";

LysosomeChannel=imagefiles[i] + " (green)";

print("... nucleus...\n");

selectWindow(“NucleusChannel”);

run("Object Counter3D", "threshold=80 slice=10 min=10 max=20971520 new_results geometrical dot=3 numbers font=12 summary");

selectWindow("Results from "+NucleusChannel);

saveAs("Results", outputdir + imagename + "_results_nucleus.xls");

run("Close");

selectWindow("Geometrical Centres "+NucleusChannel);

close();

selectWindow(NucleusChannel);

close();

print("... lysosome...\n");

selectWindow(LysosomeChannel);

run("Object Counter3D", "threshold=40 slice=10 min=10 max=20971520 new_results geometrical dot=3 numbers font=12 summary");

run("3D Watershed", "seeds_threshold=1 image_threshold=20 image=" + imagename + " seeds=Geometrical radius=2");

selectWindow("watershed");

run("Properties...", "channels=1 slices=n frames=1 unit=micron pixel_width=0.169 pixel_height=0.169 voxel_depth=0.169 frame=[0 sec] origin=0,0");

run("3D Manager Options", "volume surface centroid_(pix) centroid_(unit) distance_between_centers=10 distance_max_contact=1.80");

run("3D Manager");

Ext.Manager3D_AddImage();

selectWindow(LysosomeChannel);

Ext.Manager3D_MultiSelect();

Ext.Manager3D_SelectAll();

Ext.Manager3D_Select(0);

Ext.Manager3D_Measure();

selectWindow("3D Measure");

saveAs("Results", outputdir + imagename + "_results_lysosomes.xls");

run("Close");

selectWindow("Results from " + LysosomeChannel);

run("Close");

selectWindow(LysosomeChannel);

close();

selectWindow("watershed");

close();

selectWindow("Geometrical Centres " + LysosomeChannel);

close();

print("... finished image.\n");

}

}

setBatchMode(false);

selectWindow ("Log");

saveAs("Text", outputdir + "Log.txt");

run("Quit");
